# Plant-microbial interactions in agriculture and the use of farming systems to improve diversity and productivity

**DOI:** 10.3934/microbiol.2017.2.335

**Published:** 2017-05-11

**Authors:** Suzanne L. Ishaq

**Affiliations:** Department of Land Resources and Environmental Sciences, Montana State University, Bozeman, MT 59717, Montana, USA

**Keywords:** bacteria, climate change, farming system, fungi, nutrient exchange, pathogens, phytohormones

## Abstract

A thorough understanding of the services provided by microorganisms to the agricultural ecosystem is integral to understanding how management systems can improve or deteriorate soil health and production over the long term. Yet it is hampered by the difficulty in measuring the intersection of plant, microbe, and environment, in no small part because of the situational specificity to some plant-microbial interactions, related to soil moisture, nutrient content, climate, and local diversity. Despite this, perspective on soil microbiota in agricultural settings can inform management practices to improve the sustainability of agricultural production.

## 1. Plant-microbial Interactions

Plant-microbial interactions can be performed through a number of direct or indirect mechanisms: nutrient transfer (stemming from vitamin or siderophore production, atmospheric nitrogen fixation, enzymatic decomposition of liter in soil, or conversion of inorganic minerals to soluble compounds, especially phosphorous), direct stimulation of growth through phytohormones (such as ethylene or indole acetic acid), antagonism towards pathogenic microorganisms, and mitigation of salt stress [1]–[5]. Recent research conducted in natural and semi-natural settings indicates that plant-induced changes to the soil microbial community structure may alter ecological processes, which, in turn, modify plant performance and community structure [6],[7],[8]. In agricultural settings, these plant-microbial interactions (a.k.a. plant-soil feedbacks) can have dramatic effects on crop yield and economic viability. A clear advantage can be seen in limiting soils where, for example, arbuscular mycorrhizal fungi can enhance phosphorus uptake in highly weathered Ultisol soils, where it is strongly limited by ligation to aluminum [9]. Moreover, evidence suggests that plants will prioritize microbial interactions depending on the growing conditions and need for different compounds [10].

### 1.1. Nutrient exchange

Plants serve as the primary source of carbon for soil microorganisms, through carbon-based root exudates produced during photosynthesis or through plant-residue inputs [6],[11]. In return, soil microorganisms contribute to the cycling of soil nutrients (i.e. carbon, nitrogen, phosphorous, calcium, magnesium, etc.) through fixation of environmental elements as well as the decomposition of biological detritus. To facilitate the efficiency of plant-microbial interactions [4],[11],[12], soil-based associations take place directly adjacent to roots, called the rhizosphere. When compared to bulk soil, rhizosphere soil demonstrates relatively more low-molecular-weight dissolved organic matter, 10 times more bacterial biomass, as well as higher diversity and abundance of other rhizospheric microorganisms such as protists, viruses, and fungi, than bulk soil [13],[14]. Owing to a complex food web and interactions, the rhizosphere expresses tremendous metabolic functions and activities that nevertheless accelerate nutrient turnover around plant roots [13],[14],[15].

Moreover, soil from the rhizosphere contained more of the bacterial quorum-sensing molecule N-acyl-homoserine lactone (AHL) [13], produced exclusively by the Proteobacteria phylum [16], indicating that larger concentrations of bacteria were to be found there. The Proteobacteria phylum contains a diverse spectrum of environmental bacteria, many of which have been found in rhizosphere soil, including in the rhizosphere of agricultural crops and weeds [17]–[20]. As the functional contribution of many soil bacteria remain presumptive, this dominance of Proteobacteria in agricultural soil may reflect their role in nutrient cycling [21], their opportunistic use of the additional moisture and nutrients in agricultural soil [22],[23],[24], or their ability to survive the selective pressures of agriculture [25].

Microbial diversity has been directly correlated to above-ground diversity in a number of agricultural and natural settings [8],[17],[26],[27],[28], as increased taxonomic diversity allows for a redundancy in microbial functionality [5],[29]. Importantly, having functional redundancy in biochemical pathways increases the resiliency of a system to chemical or physical disturbance [30]–[33]. Interestingly, microbial density was shown to non-linearly affect plant production. While increasing microbial density has been shown to increase plant biomass [17],[29],[34],[35], at very high microbial density plant biomass decreased even though photo-assimilation was increased [36], indicating a more efficient plant.

### 1.2. Direct growth promotion

In addition to sharing nutrients, microorganisms take an active role in the growth of specific species using hormone production. Indoleacetic acid, or indole-3-acetic acid, is a plant hormone produced in the apex or buds and new leaves of young plants. In the auxin class, IAA and other auxins promote plant growth, specifically through cell division and elongation. However, IAA is not specific to plants and is produced by a number of bacterial species using tryptophan [37]–[40]. *Pseudomonas putida* promoted root development in canola and mung beans using IAA [37]. The bacteria *Exiguobacterium homiense*, *Bacillus pumilus*, and *Bacillus licheniformis* have also been shown to promote bud formation in red algae (*Gracilaria dura*) using IAA, as well as ammonium production [41]. *Bacillus subtillis* promoted root growth and new shoots in lettuce using IAA, abscisic acid (ABA), and several cytokinins, including zeatin riboside (ZR), dihydrozeatinriboside (DHZR) and isopentenyladenosine (iPA) [40]. However, IAA has been produced by pathogenic bacteria, as by *Agrobacterium tumefaciens* to induce tumors in plants [42],[43].

Ethylene is another plant hormone that promotes maturation of fruit, as well as induces seed germination, in a pathway that promotes cyanohydrin or nitrile production that in turn stimulates IAA production. Ethylene is a gaseous hormone produced by plants using 1-aminocyclopropane-1-carboxylate (ACC), an amino acid precursor [44]. Several fungal species have been shown to produce ethylene, including *Penicillium cyclopium* and *P. crustosum* [45], as well as the bacteria *Pseudomonas syringae* [46], allowing direct control of plant growth. On the other hand, microorganisms have also been shown to modulate ethylene production by the plant itself, using ACC deaminase enzymes to control the availability of the ACC precursor [2],[44],[47]. This has been shown to reduce salt-induced growth-retardation in plants [48].

It has been previously suggested that during forest fires, vegetation may release ethylene into soil in order to promote seed germination and regrowth [49]. However, under stressful growth conditions (ex. drought, salinity, water logging, heavy metal concentration) an abundance of ethylene can also retard root growth, which can aid in long-term survival until conditions improve [50]. Salt-stress disrupts the osmotic balance of plants, can slow plant growth, and cause cell death, all of which reduce plant productivity. While it is clear that ethylene is produced by plants in response to salt stress, there is conflicting evidence for ethylene both as a stimulant for salt-tolerance, as well as an over-reaction causing salt-sensitivity in plants (reviewed in [50],[51]). However, these differential results may be attributed to differences in soil bacterial concentration, and plant genetics. For example, many bacteria only present certain attributes when in biofilms or high concentrations, mediated by quorum sensing molecules produced when bacteria are in close proximity and which trigger RNA transcriptional changes. Genetically modified tobacco [52] and tomato plants [53], which were able to produce the quorum sensing molecule N-acyl-homoserine lactone (AHL) [16], were able to stimulate gene expression in soil bacteria, which in turn improved plant growth under salt-stress conditions. However, that modification of growth in the transgenic tomatoes was specific to whether that plant could produce either short-chain or long-chain AHL, and which probiotic bacterial strain was used [53].

### 1.3. Disease state and pest control

In addition to mitigating stress effects, plant-microbial interactions influence plant disease state or diversity of soil pathogens. For example, fungal endophytes, those living inside plant host tissue, have been shown to reduce herbivory by insects [54],[55]. Similarly, bacterial endophytes also induce resistance in host plants and increase their performance under insect herbivory [56]. A large number of interactions and studies; however, are focused on biological control of fungal pathogens. This is due to the large number of fungal pathogens, the economic difficulty of pathogen control and lost harvest, the accumulation of mycotoxins which are health hazards to humans and livestock, and the difficulty in targeting fungal contamination due to their tough cell walls, slow growth, sporulation, and ubiquitous distribution in terrestrial or aquatic sources.

A number of plant defensins (small, biologically-active peptides) have been identified with antifungal or, less commonly, antibacterial properties, as well as conferring drought-resistance, and playing a role in plant cell-signaling pathways [57]. Notably, most of these defensins have been reported in seeds, many of which disrupt microbial cell membranes or interact with them to cause internal signaling cascades that trigger fungal cell death [57],[58],[59]. Plant defensins are often inactivated by high concentrations of cations (ex. calcium or magnesium), hypothesized to be due to a change in electrostatic affinity between molecules [57]. The possibility exists; however, that this theoretical “back door” could be exploited by soil fungi as a bargaining tool in soils where calcium or magnesium are limited (i.e. vegetable farms [60]), as these cations are integral to plant growth [61], and have been shown to mediate plant-symbiont cell signaling in rhizobia [62].

More recent work has identified RNA-based mechanisms of disease stimulation by fungi or control by plants. Small RNAs are non-coding sections of RNA less than 250 bases in length and typically 20–30 bases long, which bind to other RNAs and block translation by ribosomes [63]. Small RNAs are classified by action: small interfering RNAs (siRNAs), microRNAs (miRNAs) and Piwi-associated RNAs (piRNAs) [63]. The fungus *Botrytis cinerea*, which causes grey mold disease in hundreds of plant species, can dampen plants' immune response using siRNAs to prevent transcription (a.k.a. silencing) of host plant genes involved in immunity [64]. The targeted plant genes include mitogen-activated protein kinases (MAPKs) [64], which coordinate cellular responses to stress signals (ex. osmotic, temperature) or the peptide group of phytocytokins which coordinate cell signaling during immune reactions [65]. The pleotropic nature of many plant genes, though, confers a functional redundancy which can protect against biotic (ex. siRNA) and abiotic challenges [66]. Plants have also been shown to use siRNAs to control fungal pathogens, and there has been some success using a topical siRNA spray to control *Fusarium* sp. on barley in growth chambers [67].

In addition to many plant-produced antifungals, there is interest in fungi-produced antifungals. For example, the fungus *Trichoderma harzianum* has been shown to control the growth of other, pathogenic fungi, as well as insect pests by using them as hosts [68], as it produces a number of chitinases and other lytic enzymes [69].

There are also many cross-domain antifungal interactions. For example, the bacteria *Bacillus amyloliquefaciens* reduced the growth of 12 fungal species (including *Alternaria panax*, *Botrytis cinera*, *Colletotrichum orbiculare*, *Penicillium digitatum*, *Pyricularia grisea*, and *Sclerotinia sclerotiorum*), *in vitro* and in trials with cucumber and pumpkin plants [70]. *Bacillus amyloliquefaciens* also reduced fungal rot in citrus [71], strawberries [72], and soybeans [73], and *B. velenzensis* reduced the infection of citrus green mold by the fungus *Penicillium digitatum* [74]. In field conditions, where continuously mono-cropped tobacco plants had eventually become infected with *Fusarium* due to pathogen build-up in soil, large-scale treatment of bacteria native to the soil habitat showed a reduction in fungal infection [75].

As with growth, disease state is affected by the AHL quorum-sensing molecule of bacteria. AHL can trigger the production of the *N*-(3-oxohexanoyl) homoserine lactone (OHHL) enzyme in the pathogen *Erwinia carotovora*, which controls the production of several enzymes and acids that allow the bacteria to degrade plant cell walls [76],[77]. However, AHL can also trigger the release of *N*-hexanoylhomoserine lactone (HHL), which controls the production of chitinase that aids in the degradation of fungal cell walls [78]. HHL also controls the production of other antifungal compounds (ex. butyrolactones, furanone, 2,4-diacetophloroglucinol (Phl)) by the bacteria *Pseudomonas aureofaciens* [79]–[83]. Notably, the antifungal metabolites of *P. aureofaciens* can control the fungi *Gaeumannomyces graminis* (var. tritici) [83], *Pythium* spp. [81],[82],[84], *Fusarium solani* and *F. oxysporum* [82], *Thielaviopsis basicola* [83], or *Phytophthora megasperma* [80],[81], all of which cause rot, especially in the roots, of a wide variety of agriculturally-important plants. A closer look at the antifungal metabolites of *P. aureofaciens* showed that compounds physically distort fungal growth [82], chemically degrade fungal cell organelles, or cause the plasmalemma cell membrane to retract [85]. While this membrane normally retracts during a fungal sporulation event, its retraction also makes the fungal cell more vulnerable to microbial attack [86].

### 1.4. Seed-microbial interactions

Beginning with observations that legume-conditioned soil could promote the growth of new legumes, the first patent for a “Rhizobium bacterial inoculation for plants” was granted in 1896 [87]. Monoculture bacterial seed coatings have shown mixed beneficial and inhibitory effects on plant seed germination and seedling growth parameters [36],[88]–[91], and the positive effects of a bacterial seed coating on wheat seed germination rate were diminished with high bacterial density [36]. Thus, the differential responses to bacterial soil probiotics may reflect differences in experimental design (i.e. concentration of bacteria applied, plant varieties, and bacterial strains used), and potentially a differing effect on plant efficiency (i.e. less root biomass was produced because plant-microbial interactions were productive and more biologically cost-effective) [36]. Moreover, multi-level seed-microbial interactions may do better promote seedling emergence and growth [4], further promoting the idea that total microbial diversity provides better results than any one interaction.

In a more targeted approach, bacteria and bacteriophages (viruses which infect bacteria) that were antagonistic to *Salmonella* were isolated from mung bean (*Vigna radiata*) and alfalfa (*Medicago sativa*) seeds, and then used as spray coating on seeds to control *Salmonella* growth in both crops, which commonly host this food-borne pathogen [92]. Despite the interest in microbial seed coatings, relatively few have been rigorously tested in the laboratory and the field. In an effort to fill a knowledge and economic niche, large-scale testing of microbial seed coating was recently publicized by several commercial agricultural research and development facilities in an effort to harness seed-microbial interactions [93].

On the other hand, seed-microbe interactions can be antagonistic. Microbial degradation of native or crop seeds can reduce productivity [94], and degradation of weed seeds has been investigated as an alternative, and organic, means of weed control [95],[96]. Microbial degradation of seeds can also allow for remineralization of nutrients into the soil [94], which would better support adult plants.

While it was long known that symbiotic or pathogenic endophytes (microorganisms living inside plant tissues) could be passed horizontally (i.e. contagiously) (reviewed in [54],[55]), it was only recently that microorganisms were discovered inside seeds where they had been transmitted vertically from parent to offspring [97]. Research suggests that vertical transmission of microorganisms or viruses selectively reduce pathogenicity and virulence, since endophytes in seeds must not negatively affect the reproductive success of their hosts [98],[99], which may lead to more symbiotic plant-microbial/viral interactions over time.

## 2. Effect of Farming System on Plant-microbial Interactions

Farming systems are broadly grouped into those which use chemical or synthetic means of pest control and nutrient fertilization (a.k.a. conventional), and those which don't in favor of an integrated system with the goal of sustainability (a.k.a. organic). Within each system, a number of management techniques may be used which collectively alter above-ground and below-ground biodiversity, including chemical use, fertilization, irrigation, crop rotation or crop-fallow rotations, co-cropping, livestock grazing, etc. A number of studies broadly comparing organic and conventional systems have shown differences in crop production, competition by weeds, pests, or microbial pathogens [100]. Notably, organic farming, and often the increased soil organic matter associated with organic farms, selected for a higher overall microbial diversity [17],[19],[101],[102],[103].

### 2.1. Soil fertilization

Soil fertilization utilizes organic matter (mulching) or chemical supplementation to add nutrients back into soil. The availability of nutrients can dramatically shift plant diversity and functionality, and fertilizers must be properly balanced to prevent “fertilizer burn”, in which over-application of salts desiccates plant structures and causes damage through osmotic stress. In much the same ways, microorganisms are sensitive to fertilization. Long-term use of mineral fertilization has been shown to increase bacterial and fungal diversity, microbial biomass carbon, as well as dehydrogenase and other enzyme activity [104].

However, these benefits are variable depending on the type and source of minerals. Using only mineral nitrogen (typically ammonium sulfate) does not increase soil microbial diversity [104],[105],[106] and may even reduce it [107]. Phosphorous-only supplementation has a similar lack of effect [106] except where it was limiting [108]. This reduction may be driven by a shift towards more acidic soil which tends to reduce total microbial diversity and shift towards acid-tolerant species, such as within the bacterial phyla Acidobacteria [25],[106],[109],[110]. It may also be a function of the relative type and amount of plant residues [111], or a change in nutrient availability and the carbon:nitrogen ratio in soil [105],[106].

Animal manure has been shown to be significantly more effective at increasing microbial biomass than mineral fertilization [103],[104]. Integrated livestock grazing has recently re-emerged as an alternative method of crop-residue removal, specifically in organic systems [112]. Its implementation has been slow, especially in large production systems, as the use of grazing livestock can be time and labor-intensive. Inputs of feces and urine from livestock grazing increases soil organic carbon and nitrogen [113], as well as total microbial biomass [113],[114]. However, this may only be reflected in bacterial biomass and not an increase in fungal biomass [115]. In systems where grazing pressure is high, this effect can be reversed as soil nutrients are lost to erosion caused by a lack of plant cover material [113],[116],[117].

### 2.2. Cover crops

Cover crops are grown as an alternative to fallowing, or leaving a field unplanted to rest. They provide additional economic benefit [118],[119], feed for livestock [120], reduce erosion, and facilitate weed and insect pest management [112],[118],[121]. Specifically, cover crops reduce weed seed production via competitive exclusion [122], or decreasing weed seed survival by recruiting a microbial community which contributes to seed decay [123],[124]. Mineralization of cover crop residues can increase soil organic matter [125],[126], which can increase cation exchange capacity, and enhance cycling of macronutrients [127].

Not only do the additional inputs of organic matter from cover crop residues encourage microbial diversity, but they allow for above-ground biomass to generate more below-ground biomass [125],[126],[128],[129],[130]. Crop rotations can also improve soil quality and microbial diversity [131]. The use of legumes as a cover crop or in rotation, or other crops which encourage rhizobial symbiotic bacteria to biologically fix nitrogen, and the subsequent mineralization of those nitrogen-rich plant residues back into the soil can provide usable available nitrogen for other plant species [130],[132]. For example, bacterial liter increased most in response to clover (*Trifolium repens* L.) conditioning compared with wheat (*Triticum aesativum* L.), ryegrass (*Lolium perenne* L.), bentgrass (*Agrostis capillaris* L.), or sucrose conditioning [133]. Additionally, microbial communities differed strongly among the four cover crop conditioning species [133].

### 2.3. Tillage

In both organic and conventional systems, tillage is the most common method of incorporating crop residues back into soil, as well as redistributing weed seeds either further into soil to prevent germination or onto the surface where they may be eaten. Due to the disruptive nature of tillage in the first 30–50 cm of topsoil, significant detriment can be done by physically destroying mycorrhizal root colonization [134]. Moreover, soil microbial diversity and density is highly correlative to soil depth and local factors (ex. oxygen content, UV light, moisture). Thus, intensive soil tillage can drastically decrease soil microbial diversity and density, specifically bacterial and fungal, through erosion and wind dispersion of microorganisms or nutrients, or through selective culling of sub-surface species brought to the surface [9],[25],[131],[135]–[138]. However, addition of soil organic matter through mulching may attenuate some of these adverse effects [131],[136]. No-till systems typically have more soil carbon [139].

### 2.4. Chemical control and bioremediation of farmland

Chemical control used for managing agricultural systems has been show to alter the microbial community, notably in decreasing diversity [140],[141],[142]. However, the persistence of pesticides and other chemical contaminates in soil is also of concern for biological systems in natural and agricultural settings, not only because they may accrue and affect other beneficial organisms and soil health indicators, but many contain heavy metals which are toxic in of themselves [143]. Additionally, local water sources and runoff may add contaminants from exogenous sources. Phyto, microbial, or combined bioremediation of chemical contamination has sought to degrade or detoxify pesticides (i.e. herbicides, insecticides, fungicides, rodenticides, etc.), heavy metals, and antibiotics.

For example, bacteria belonging to the genera *Acinetobacter*, *Alcaligenes*, *Arthrobacter*, *Bacillus*, *Burkholderia*, *Corynebacterium*, *Flavobacterium*, *Micrococcus*, *Mycobacterium*, *Pseudomonas*, *Sphingomonas*, and *Rhodococcus*, and the fungus *Phanerochaete chrysosporium* are just of few of the microorganisms shown to degrade different types of hydrocarbons from petroleum spills [144]–[148]. The degradation of chemicals, the sequestration of heavy metals, or the detoxification of heavy metal compounds by microorganisms is dependent on the nature of the compound, as well as the ambient conditions of the environment [145],[149]. Endosulfan degradation depends on soil type and oxygen content [150],[151], as well as soil texture, organic matter content, inoculum concentration, pH, and specificity of bacterial strains used [147]. Similarly, dichlorodiphenyltrichloroetano (DDT), metoxychlor, and gamma-hexachlorociclohexane (gamma-HCH) degradation processes are dependent on temperature [152]. HCH degradation was also shown to be dependent on oxygen content and nitrate concentration [153]. An additional nutrient source, such as molasses, is often needed to increase the rate of chemical degradation in culture [154],[155].

Field trials have been focused on removing chemical and metal contamination from either soil or water runoff, either using direct application of microorganisms or the use of a “biobed” as a biological filter or retaining system to remove contaminants from farm waste water [156]. The bacteria *Mycobacterium gilvum* was successfully used to degrade polycyclic aromatic hydrocarbons, and increase soil bacterial diversity, on a vegetable farm [157]. A strain of *Arthrobacter* and another of *Bacillus* were used to reduce metal contamination in soil, improve rice biomass production, and reduce the amount of metal accumulated in rice [158]. Halophilic bacteria were used to remove salt left behind after the March 2011 tsunami in Japan, as well as green compost to restore organic matter that had been washed away [159]. Furthermore, bacteria that are able to mitigate salt-stress in plants can promote growth into similarly affected areas [51],[160].

The concept of remediating soil diversity towards a “more natural” community has been slower to take root. A study of pre-agricultural prairie soil reported a very different bacterial community than that found in human-associated agricultural soil [25]. Notably, prairie soils were dominated by the bacterial phylum Verrucomicrobia, whereas agricultural soil shows a dominance of Proteobacteria, Bacteroidetes, or Firmicutes [17],[101]. Verrucomicrobia grow more slowly, but survive better in nutrient-limiting soils. Likewise, Acidobacteria are also known to survive under nutrient-limiting (oligotrophic) conditions [24],[161],[162],[163]. Moreover, Verrucomicrobia from pre-agricultural soil contained more genes for carbohydrate metabolism than nitrogen metabolism [25], suggesting that their abundance in agricultural soil may be negatively selected for by the use of nitrogen fertilizer. And, as Proteobacteria produce the quorum-sensing molecule AHL which triggers beneficial and pathogenic responses from bacteria, selecting for these species under agricultural conditions may be contributing to plant disease dynamics.

## 3. Climate Dynamics

Changing climate poses problems to global food production as atmospheric gas concentrations, temperatures, seasonal growing days, water availability and soil moisture, extreme weather events, and pest populations are changing. Research into the effects of increased carbon dioxide (CO_2_) on plants has shown that plants initially fare better under increased CO_2_: biomass and cellular respiration are increased. This increase in plant production depletes the soil of organic matter, carbon, nitrogen, and moisture [164]. After several years under increased CO_2_, plant growth and respiration slow as the plant acclimates to the new conditions, at which point biomass production returns to pre-treatment levels or below [165],[166].

However, an increase in biomass does not necessarily translate to an increase in production or nutritional value, if that biomass increase is strictly structural carbohydrates. Following increased CO_2_, potassium, zinc, iron, nitrogen, and protein have been reduced [167]–[171] in a variety of agricultural crops. Isoflavone, which is under investigation as a dietary estrogen-analogue, was reduced in soybeans [172]. Potassium, a production-limiting nutrient in cotton production, was also reduced [173].

The increase in plant biomass allows for increased shading, which can decrease soil temperature even as air temperature is increased. This drop in temperature can slow microbial growth and function, such that decomposition of biological detritus in soil is reduced [132],[164],[174],[175],[176], and cannot replace what the faster-growing plants are removing. Microbial functionality can also be decreased when soil temperature is increased above 30 °C [174],[176],[177],[178], or when temperature fluctuates [179]. Moreover, temperature selects for different bacterial and fungal diversity, which will also drive different community-wide enzymatic abilities [180].

In addition to drought-tolerant or heat-resistant crop varieties, it may be possible to condition soil towards a more drought-tolerant microbial community, or one that can better withstand changing soil temperatures. Steam pasteurization of soils has shown a temporary but recoverable decrease in microbial activity [181], indicating a degree of flexibility. Fungal diversity and mycorrhizal growth has also been implicated in improving water use efficiency, possibly by moderating the exchange of nutrients with roots and the resulting osmotic pressure changes [182]. While drought conditions have previously increased microbial biomass in soil, it also prompted a reduction in plant-based carbon sequestration into soil via roots exudates, specifically to bacterial targets but not to fungal symbionts [10]. In persistent drought conditions, long-term reductions on soil carbon and a shift in bacterial diversity may eventually feedback negatively to above-ground diversity and production.
